# Chronic inorganic arsenic exposure impairs locomotor performance and socio-affective behavior in zebrafish

**DOI:** 10.17305/bb.2026.14094

**Published:** 2026-04-10

**Authors:** Cătălina Ionescu, Petru Fabian Lungu, Ionut-Alexandru Chelaru, Mircea Nicusor Nicoara, Gabriel Plavan, Ioana-Miruna Balmus, Viorica Rarinca, Alin Ciobica, Bogdan Novac, Diana Gheban, Daniela-Ivona Tomiţa, Alexandra Savuca, Bogdan Gurzu

**Affiliations:** 1 Doctoral School of Biology, Faculty of Biology, “Alexandru Ioan Cuza” University of Iasi, Iaşi, Romania; 2 “Ioan Haulica” Institute, Apollonia University, Iaşi, Romania; 3 Department of Biological and Morphofunctional Sciences, College of Medicine and Biological Science, Stefan cel Mare University of Suceava, Suceava, Romania; 4 Department of Biology, Faculty of Sciences, University “Vasile Alecsandri” of Bacau, Bacau, Romania; 5 Doctoral School of Geosciences, Faculty of Geography and Geology, “Alexandru Ioan Cuza” University of Iasi, Iaşi, Romania; 6 Department of Biology, Faculty of Biology, Alexandru Ioan Cuza University of Iasi, Iaşi, Romania; 7 Department of Exact Sciences and Natural Sciences, Institute of Interdisciplinary Research, Alexandru Ioan Cuza University of Iasi, Iaşi, Romania; 8 “Olga Necrasov” Center, Depart. of Biomedical Research, Romanian Academy, Iaşi, Romania; 9 Academy of Romanian Scientists, Bucharest, Romania; 10 CENEMED Platform for Interdisciplinary Research, “Grigore T. Popa” University of Medicine and Pharmacy, Iaşi, Romania; 11 Faculty of Medicine, University of Medicine and Pharmacy “Grigore T. Popa”, Iaşi, Romania; 12 Department of Dental Medicine, Apollonia University, Iaşi, Romania

**Keywords:** Arsenic, zebrafish, toxicity assessment, arsenic exposure

## Abstract

Inorganic arsenic (iAs) is a prevalent and highly toxic environmental contaminant, yet its neurotoxic effects on aquatic organisms are not well characterized. This study examines whether chronic exposure to environmentally relevant concentrations of iAs affects locomotor performance and socio-affective behavior in adult zebrafish (*Danio rerio*). Zebrafish were exposed to 10, 50, or 300 µg/L of iAs for 21 consecutive days, with behavioral assessments conducted on Days 0, 7, 14, and 21 using standardized locomotor and social preference tasks. Chronic exposure to iAs resulted in time- and dose-dependent neurobehavioral alterations. The most pronounced locomotor effects occurred at concentrations of 50 and 300 µg/L, revealing significant changes in swimming velocity, immobility duration, and counter-clockwise rotations, which indicate disrupted swimming patterns and altered decision-making behavior. Additionally, in the social task, chronic iAs exposure diminished social preference, evidenced by reduced time spent in the social arm and broader shifts in maze-arm exploration, highlighting a dose-dependent disruption of socio-affective behavior. Overall, chronic exposure to environmentally relevant concentrations of iAs is linked to neurobehavioral impairments that affect locomotion, exploratory and decision-making behavior, and social interactions in zebrafish. These findings underscore the neurotoxic potential of arsenic in aquatic organisms and emphasize surface-water contamination as a significant ecological and public health concern.

## Introduction

Being a naturally occurring semi-metallic compound, arsenic (As) is widely distributed across various environmental types on Earth, ranking as the 20th most abundant element in soils and the 14th in marine environments [1–3]. Inorganic arsenic (iAs) primarily exists in three forms: metalloid arsenic (As (0)), arsenite (As (III)), and arsenate (As (V)), with the latter two forms predominating in natural and drinking water sources [2]. Under mildly reducing conditions, As (III) is significantly more toxic than As (V) due to its greater reactivity with cellular components [4]. Inefficient soil adsorption and increased mobility of As (III) complicate water treatment efforts aimed at mitigating its accumulation [5, 6]. The extensive and prolonged use of arsenic-based herbicides, pesticides, and wood preservatives has significantly contributed to environmental arsenic accumulation [7].

The dual metallic and non-metallic properties of iAs classify it as a notorious agent of damage to human skin and lungs, with potential carcinogenic effects as noted by the International Agency for Research on Cancer (IARC) [3]. Additionally, both humans and aquatic organisms are particularly susceptible to unavoidable exposure to iAs, with its accumulation in various organs serving as a common indicator of metallic pollution in aquatic ecosystems [8]. Previous studies have indicated that iAs can accumulate in muscle tissues and disrupt immune, respiratory, digestive, excretory, reproductive, nervous, and endocrine systems [8, 9].

Several studies have reported severe effects of iAs exposure on cognitive functions in both humans and rodents, including impairments in learning, memory processes, and significant contributions to mood disorders [10, 11].

Zebrafish represent a valuable species for neurobehavioral research due to their genetic and behavioral similarities to humans. They exhibit a wide range of neurobehavioral patterns, including neuroplasticity, memory, learning, neuro-affective responses, and social behavior [12]. Zebrafish, in particular, display mammalian-like physiological responses and behavioral abnormalities when exposed to environmental pollutants [13]. An important similarity between zebrafish and mammalian species is the presence of a highly specialized blood-brain barrier (BBB), which is crucial for regulating the access of blood constituents to brain tissues [14]. Previous research has linked arsenic exposure to neurotoxicity, particularly cognitive and neurobehavioral impairments, through effects on neurotransmission and brain monoamine synthesis in various animal models [15]. Chronic exposure to environmentally relevant arsenic doses in zebrafish has been associated with neurobehavioral changes, alterations in the dopamine system, and endocrine disruptions [16]. Additionally, while the BBB of zebrafish closely resembles that of mammals, some studies have indicated that arsenic can easily traverse this barrier and accumulate within brain structures [17].

In this context, this study aims to evaluate neurobehavioral changes resulting from chronic exposure to iAs in zebrafish, an animal model of significant relevance to research. The objectives include assessing behavioral patterns related to general cognitive performance and socio-affective contexts that may be impaired by chronic iAs exposure at environmentally relevant concentrations.

## Materials and methods

### Animal maintenance

Forty adult zebrafish (approximately 10–11 months old) with a balanced sex ratio (1:1 male to female) and weighing between 180 and 220 mg were obtained from an authorized local breeder. The zebrafish were acclimatized to laboratory conditions for 10 days in 10 L aquariums filled with dechlorinated water, with daily water replacement. The temperature was maintained at 26 ± 1 ^∘^C, pH at 7.5, dissolved oxygen at 7.20 mg/L, ammonia concentration below 0.004 ppm, and conductivity at 500 µS. The acclimatization aquaria were subjected to a light/dark cycle of 12:12 h (8 am/pm switch points). The animals were fed daily with standard fish formulations (Tetramin Flakes, Germany).

The care and handling of the animals adhered to the EU Commission Recommendations (2007), Directive 2010/63/EU of the European Parliament, and Council guidelines of 22 September 2010 on the accommodation, care, and protection of animals used for experimental and other scientific purposes. All procedures involving the animals were performed in accordance with the recommendations of the local Ethics Committee (Faculty of Biology, “Alexandru Ioan Cuza” University of Iasi, registration no. 5585/12.12.2024).

### Experimental design

Following acclimatization, the zebrafish were randomly assigned to four experimental groups (*n* ═ 10/group). Each group was housed in a single tank, with all individuals within a tank subjected to the same treatment, thereby representing the true experimental unit for this study.

One group served as the control, while the other three groups were exposed to inorganic arsenic via a certified arsenic standard solution (H_3_AsO_4_ in 0.5 M HNO_3_, 1000 mg/L As, Sigma-Aldrich, Germany), which is traceable to National Institute of Standards and Technology (NIST) standards. The nominal exposure concentrations (10, 50, and 300 µg/L) were selected based on reported levels in environmental surface water and relevant ecotoxicological studies [18]. Freshly prepared solutions were added daily to the acclimatization tanks over a 21-day period, following standard water replacement procedures. During the exposure period, water quality was regularly monitored, maintaining pH at 7.5 ± 0.2 to ensure physiological conditions were preserved throughout the experiment.

### Behavioral analysis

Behavioral responses were analyzed at four key time points during the chronic exposure period (Day 0, Day 7, Day 14, and Day 21). Social-affective behavior and locomotor performance of the zebrafish were evaluated using standardized behavioral tasks. All video captures were analyzed using EthoVisionXT 16 video tracking software (Noldus Information Technology, Wageningen, The Netherlands). A detailed schematic of the experimental design is presented in Figure 1.

**Figure 1.**
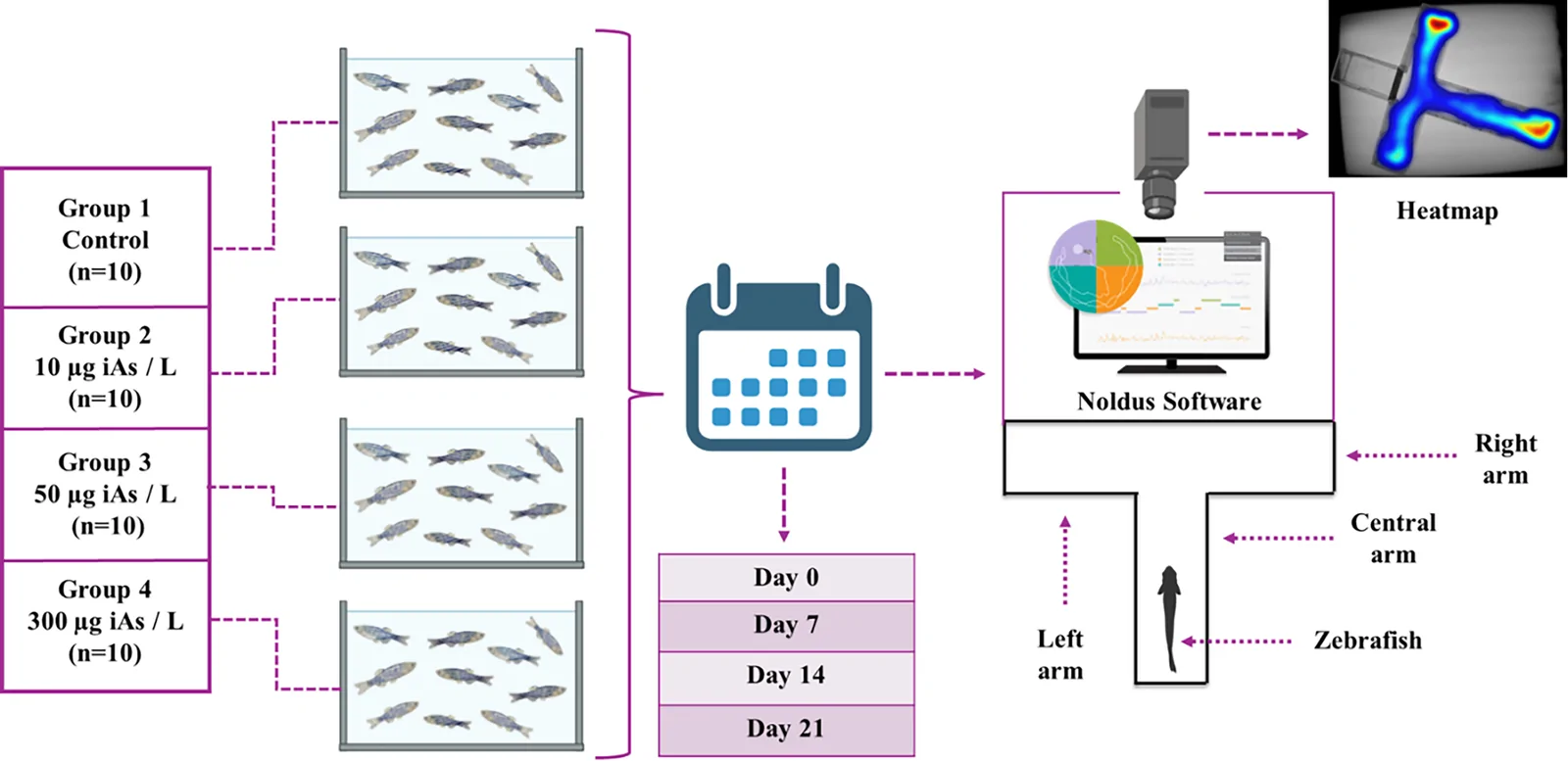
**Schematic overview of the experimental design used to assess the behavioural effects of chronic inorganic arsenic exposure in adult zebrafish.** Adult zebrafish (*Danio rerio*) were randomly allocated to four experimental groups (10 fish per group): Control, 10, 50, and 300 µg/L iAs. Each group was maintained in a separate exposure tank and monitored over a 21-day exposure period. Behavioural assessments were performed at four predefined time points (Days 0, 7, 14, and 21). As illustrated, locomotor performance and socio-affective behaviour were evaluated in a T-maze–based setup with overhead video acquisition, and the recorded trajectories were analysed using EthoVision XT 16 software; a representative trajectory heat map is shown. *The figure was created in part with BioRender.com.* **Abbreviation:** IAs, inorganic arsenic.

#### Locomotor performance test

The locomotor performance test utilized a standard T-maze setup, following previously established methodologies [19–21]. Locomotor performance was observed for 5 min, evaluating movement patterns, exploratory behavior, and decision-making behaviors through behavioral parameters available via EthoVisionXT software analysis (total swimming distance, swimming velocity, immobility time, and counterclockwise rotations). The potential significance of total immobility time as an indicator of anxiety-like behavior has been documented [22]. Similarly, the frequency of counterclockwise rotations has been identified as relevant in assessing decisional behavior [20]. All trials were conducted under standardized conditions, with water temperature maintained at 26 ± 1 ^∘^C and lighting at approximately 200 lux.

#### Social preference test

The social preference test was conducted using a T-maze setup, adhering to previously established methodological guidelines [23]. Social behavior was assessed during a 5-minute session in which the left arm of the maze contained a group of three conspecifics, separated from the test fish by a transparent wall to allow visual and olfactory cues without physical contact. Test animals were consecutively introduced into the starting arm and allowed to explore the maze freely. Zones were defined as the starting arm, left (social) arm, and right (empty) arm, with behavioral endpoints operationally defined as left arm entry frequency, total distance moved, and average velocity. Time spent in the social arm (left arm) was considered the primary outcome for social preference, while the other endpoints were analyzed descriptively to explore behavioral trends. Swimming performance relative to the social environment was assessed using these parameters, tracked with EthoVision XT. All trials were conducted under standardized conditions, with water temperature maintained at 26 ± 1 ^∘^C and lighting at approximately 200 lux. Given the multiple endpoints and time points, these analyses are considered exploratory, and findings are interpreted cautiously as indicative of behavioral trends rather than definitive effects.

### Statistical analysis

All numerical data were statistically analyzed using GraphPad Prism 9 software (San Diego, CA, USA). Data normality and distribution were assessed using the Shapiro–Wilk test. In the context of a repeated-measures design (with assessments on Days 0, 7, 14, and 21 in the same individuals), time was treated as a within-subject factor, while iAs concentration was treated as a between-subject factor. Two-way analysis of variance (ANOVA) was employed to evaluate the effects of time and iAs concentration on the time spent in maze arms for Days 7, 14, and 21, followed by Sidak’s post hoc test. While the analysis was conducted at the level of individual fish, results should be interpreted with caution, as fish within the same tank were not fully independent.

For the social-preference analysis, time spent in the social arm (Left Arm) at Days 7, 14, and 21 was considered the primary outcome and was analyzed using two-way repeated-measures ANOVA. Secondary social endpoints, including total distance moved, average velocity, and left arm entry frequency, were analyzed descriptively to explore behavioral trends. Given the multiple endpoints and time points, these analyses are considered exploratory, and results should be interpreted cautiously as indicative of behavioral trends rather than definitive effects.

Considering the exploratory nature of the study, results were interpreted with caution, focusing on consistent behavioral trends. Time spent in maze zones was treated as compositional data and primarily interpreted descriptively. Results are presented as mean ± standard deviation (SD), with statistical significance set at *P* < 0.05.

Although a balanced sex ratio (1:1) was maintained, sex was not included as a factor in the statistical analysis, which is acknowledged as a limitation of the study.

## Results

### Locomotory performance

Following chronic exposure to 10, 50, and 300 µg iAs/L, notable changes in swimming patterns of the animal models were observed. While the distance moved was not significantly affected (Figure 2A), a significant increase in swimming velocity was noted after 21 days of exposure to 50 µg iAs/L (*P ═* 0.04, vs Day 0) (Figure 2B). Significant changes in total immobility time, a potentially anxiety-relevant parameter, were observed after 21 days of exposure to 300 µg iAs/L compared to earlier assessment points (*P* ═ 0.049, vs Day 7) (Figure 2C). Moreover, the frequency of counterclockwise rotations was significantly altered by exposure to 300 µg iAs/L, particularly when comparing assessment points of Days 14 and 21 (*P* ═ 0.025) (Figure 2D).

**Figure 2.**
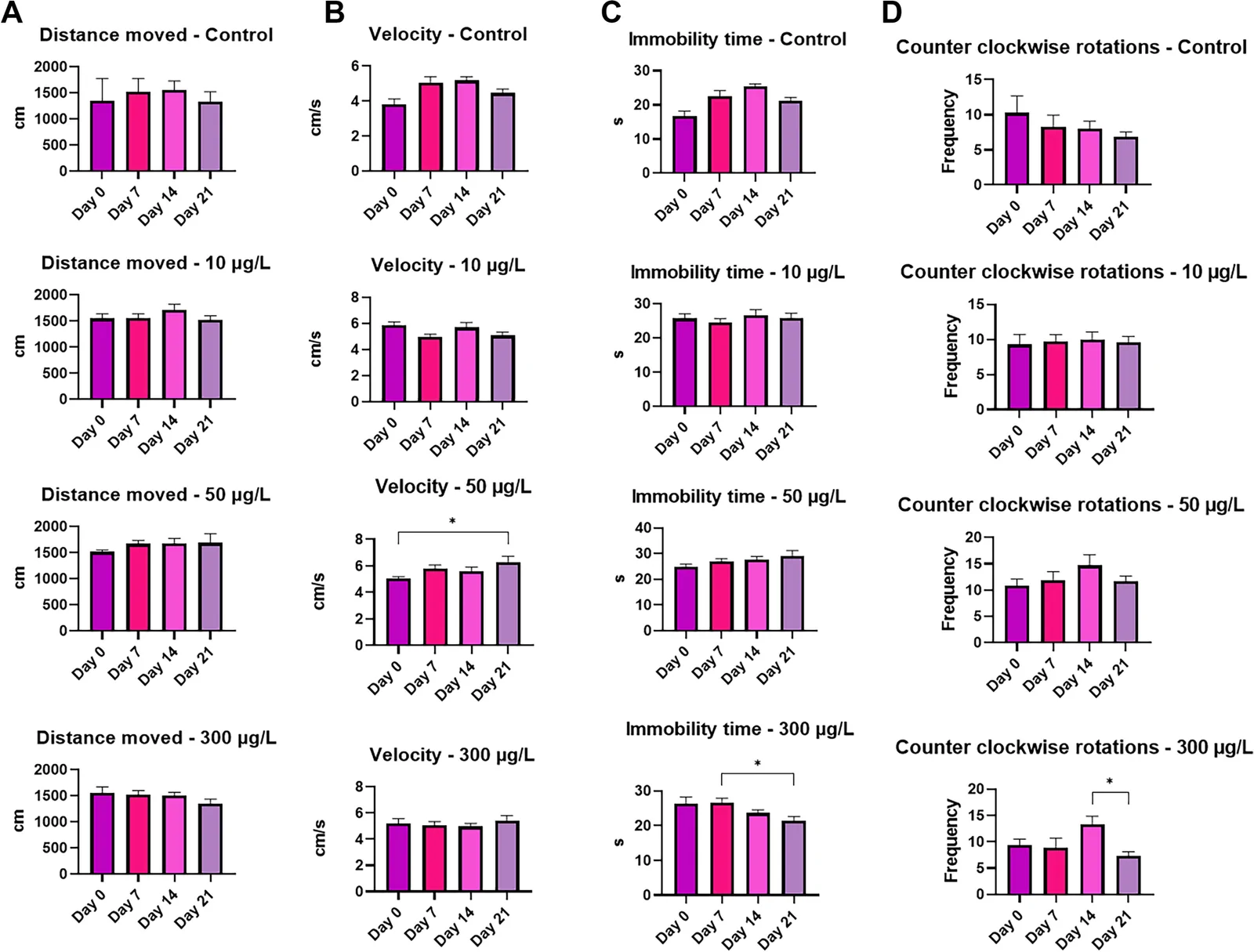
**Temporal changes in locomotor performance in adult zebrafish during chronic inorganic arsenic exposure.** (A) Total distance moved (cm) in control zebrafish and zebrafish exposed to 10, 50, or 300 µg/L iAs, assessed on Days 0, 7, 14, and 21; no significant differences were observed in this parameter during the exposure period. (B) Swimming velocity (cm/s) measured across the same time points and exposure groups; a significant increase was observed after 21 days of exposure to 50 µg/L iAs compared with Day 0. (C) Immobility time (s), used as an indicator of altered locomotor and anxiety-like behaviour, showing a significant change after 21 days of exposure to 300 µg/L iAs compared with Day 7. (D) Counterclockwise rotations (frequency), reflecting altered exploratory and decision-related behaviour, with a significant difference observed in the 300 µg/L iAs group between Days 14 and 21. Data are presented as mean ± SD (*n* ═ 10 fish/group). *P < 0.05. **Abbreviations:** IAs, inorganic arsenic; SD, standard deviation.

Regarding the relationship between dose and exposure duration in determining neurobehavioral responses, significant variations in locomotory impairments and anxiety-like behavioral patterns were observed after 21 days in relation to exposure doses (Figure 3). Specifically, swimming velocity significantly increased after 21 days of exposure to 50 µg iAs/L compared to control and 10 µg iAs/L (*P* < 0.001 and *P* ═ 0.028, respectively) (Figure 3B). Similar variation patterns were evident for total immobility time, with 50 µg iAs/L inducing peak levels for this behavioral parameter (*P* < 0.001, vs control and 300 µg iAs/L) (Figure 3C). Additionally, 14 days of exposure to 50 µg iAs/L were sufficient to elicit peak variations in counterclockwise rotations (*P* ═ 0.004 vs control, and *P* ═ 0.038 vs 300 µg iAs/L) (Figure 3D).

**Figure 3.**
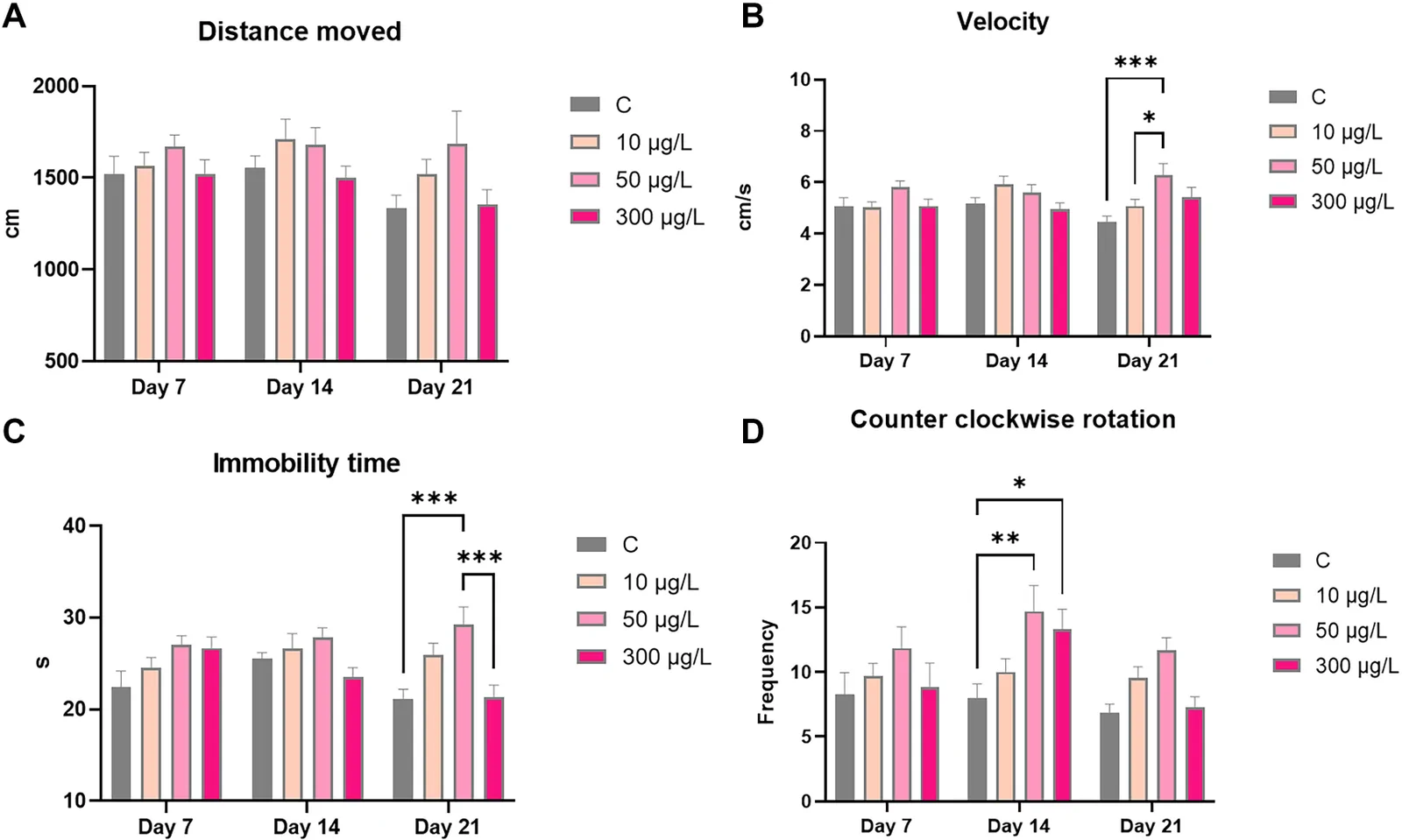
**Dose- and time-dependent effects of chronic inorganic arsenic exposure on locomotor performance in adult zebrafish.** (A) Total distance moved (cm) at Days 7, 14, and 21 in control zebrafish and zebrafish exposed to 10, 50, or 300 µg/L iAs, showing modest dose-related variation without statistically significant differences. (B) Swimming velocity (cm/s) across the same exposure groups and assessment points, with a significant increase in the 50 µg/L iAs group on Day 21 compared with the control and 10 µg/L iAs groups. (C) Immobility time (s), presented as an indicator of altered locomotor activity and anxiety-like behaviour, showing the highest values in the 50 µg/L iAs group on Day 21, significantly different from the control and 300 µg/L iAs groups. (D) Counterclockwise rotations (frequency), reflecting altered exploratory and decision-related behaviour, with a prominent peak in the 50 µg/L iAs group on Day 14 compared with the control and 300 µg/L iAs groups. For each panel, data are shown for Days 7, 14, and 21, and within each time point the bars represent the control, 10, 50, and 300 µg/L exposure groups. Results are presented as mean ± SD (*n* ═ 10 fish/group). *P < 0.05, **P < 0.01, ***P < 0.001. **Abbreviations:** IAs, inorganic arsenic; SD, standard deviation.

### Socio-affective behavior

The primary measure of social preference was the time spent in the social arm (Left Arm) at Days 7, 14, and 21, analyzed using two-way repeated-measures ANOVA (time × dose). Other behavioral endpoints, including total distance moved, average velocity, and left arm entry frequency, were analyzed using one-way ANOVA; these analyses are considered exploratory, and results should be interpreted cautiously as indicative of behavioral trends rather than definitive effects.

Significant differences in exploratory behavior were observed after 14 days of exposure to 10 µg iAs/L (*P ═* 0.019, compared to Day 0) (Figure 4). Conversely, exposure to 300 µg iAs/L for 21 days resulted in a significant decrease in total distance moved (*P ═* 0.022 vs control, and *P ═* 0.03 vs Day 7) (Figure 4A). Swimming velocity in a social context was also significantly affected after exposure to 10 µg iAs/L for 14 days, compared to 7 days of exposure (*P ═* 0.037) (Figure 4B). A peak difference was observed in the social preference parameter (left arm entry) when models were exposed to 50 µg iAs/L between Days 7 and 14 (*P ═* 0.012) (Figure 4C).

**Figure 4.**
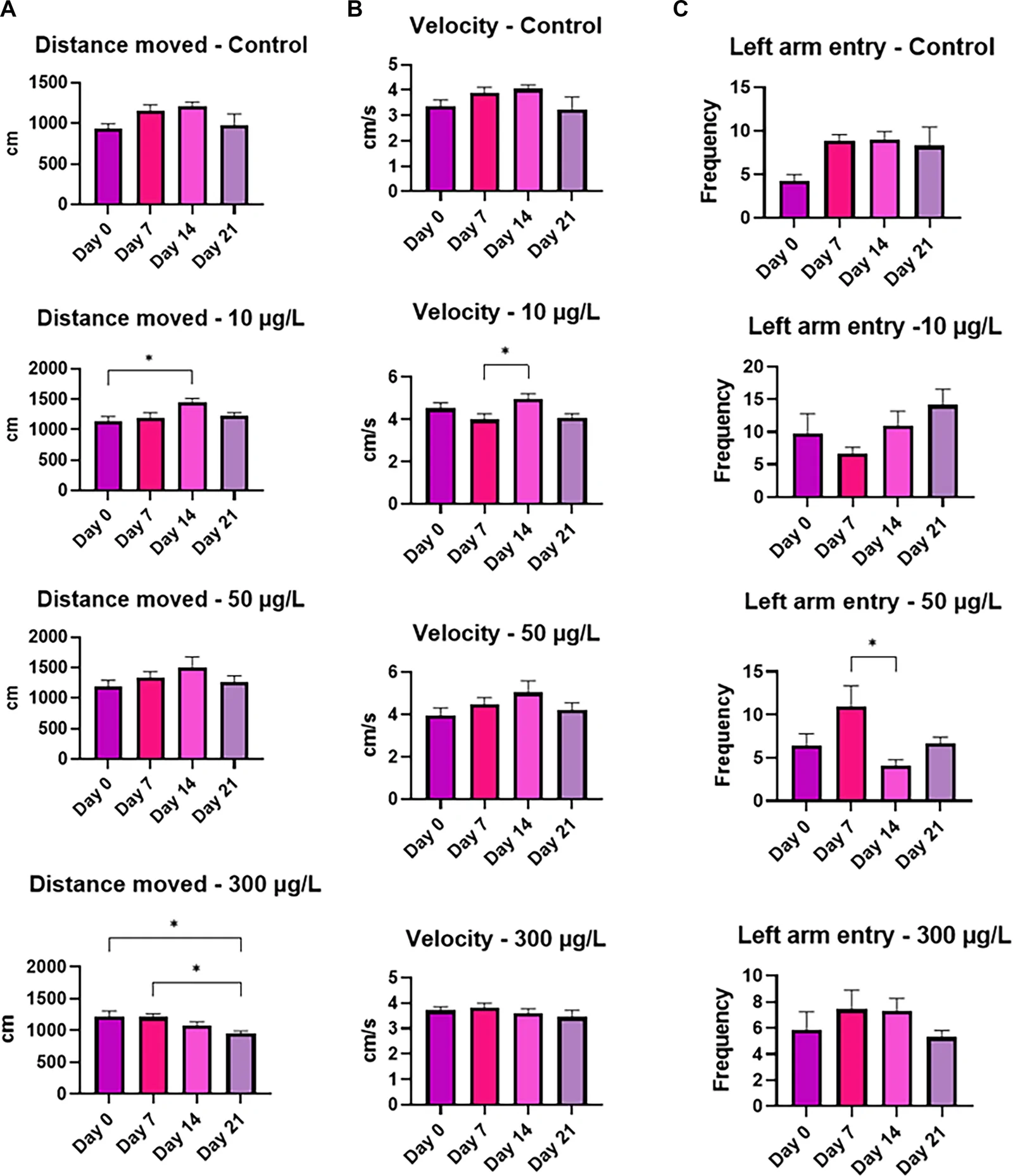
**Exploratory socio-affective behavioural parameters measured during the Social Preference Test in adult zebrafish during chronic inorganic arsenic exposure.** (A) Total distance moved (cm) in the social-preference apparatus at Days 0, 7, 14, and 21 in control zebrafish and zebrafish exposed to 10, 50, or 300 µg/L iAs; a significant decrease was observed after 21 days in the 300 µg/L iAs group compared with the control group and with Day 7. (B) Swimming velocity (cm/s) measured under the same testing conditions, showing a significant change in the 10 µg/L iAs group at Day 14 compared with Day 7. (C) Left-arm entry frequency, reflecting exploratory approach to the arm containing conspecific social stimuli, with a significant difference in the 50 µg/L iAs group between Days 7 and 14. In each column, rows correspond to the four assessment time points (top to bottom: Days 0, 7, 14, and 21), and bars within each plot represent the control, 10, 50, and 300 µg/L iAs groups, respectively. These variables were analysed as exploratory secondary endpoints of the social-preference assay. Data are presented as mean ± SD (*n* ═ 10 fish/group). Statistical significance is indicated as *P < 0.05, **P < 0.01, and ***P < 0.001. **Abbreviations:** IAs, inorganic arsenic; SD, standard deviation.

Additionally, when comparing the balance between time spent in the left arm and other arms, a significant increase in time spent in the right arm was noted after 7 days of exposure to all iAs concentrations, compared to the control group (p10 µg iAs/L = 0.007, p50 µg iAs/L = 0.049, and p300 µg iAs/L = 0.003) (Figure 5A). A visible and progressive decrease was recorded in time spent in the left arm up to 50 µg iAs/L, alongside a progressive increase in time spent in the decision point. Furthermore, a progressive visual decrease in time spent in the starting arm was observed (Figure 5A).

**Figure 5.**
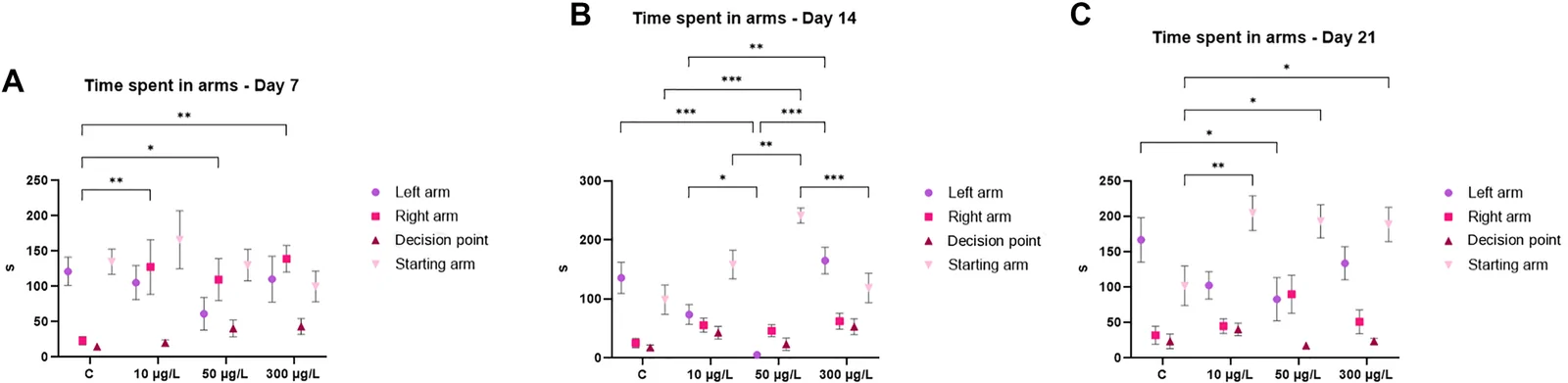
**Zone-specific occupancy in the social preference apparatus at Days 7, 14, and 21 of chronic inorganic arsenic exposure in adult zebrafish.** (A) Day 7: Time spent in the left arm (social arm; primary social-preference outcome), right arm (empty arm), decision point, and starting arm in control zebrafish and zebrafish exposed to 10, 50, or 300 µg/L iAs. At this time point, all iAs-exposed groups showed significantly increased time spent in the right arm compared with the control group, together with a progressive reduction in left-arm occupancy up to 50 µg/L. (B) Day 14: Distribution of time spent across the same apparatus zones, showing a significant decrease in time spent in the left arm in the 50 µg/L group compared with the control and 10 µg/L groups, as well as a significant increase in time spent in the starting arm, consistent with altered social preference and behavioural decision-making. (C) Day 21: Zone occupancy after prolonged exposure, demonstrating a significant reduction in time spent in the left arm in the 50 µg/L group versus control, together with significantly increased time spent in the starting arm in all iAs-exposed groups relative to the corresponding control group. Data are presented as mean ± SD (*n* ═ 10 fish/group). Statistical significance is indicated as *P < 0.05, **P < 0.01, and ***P < 0.001. **Abbreviations:** IAs, inorganic arsenic; SD, standard deviation.

During the behavioral assessment on Day 14 of exposure, a significant decrease was recorded in time spent in the left arm between 10 µg iAs/L and 50 µg iAs/L (*P ═* 0.036), between the control group and 50 µg iAs/L (*P <* 0.001), and between 10 µg iAs/L and 300 µg iAs/L (*P ═* 0.002), alongside an increase between 50 µg/L and 300 µg/L (*P <* 0.001) (Figure 5B). A progressive and significant increase in time spent in the starting arm was noted between the control group and 50 µg iAs/L (*P <* 0.001), as well as between 10 µg iAs/L and 50 µg iAs/L (*P ═* 0.003), and a decrease between 50 µg iAs/L and 300 µg iAs/L (*P <* 0.001).

At the conclusion of the exposure design on Day 21, a significant decrease in time spent in the left arm was recorded after exposure to 50 µg iAs/L (*P ═* 0.041, vs control) (Figure 5C). Significant increases in time spent in the starting arm were observed after exposure to all iAs concentrations for 21 days, compared to the corresponding control group (p10 µg iAs/L = 0.004, p50 µg iAs/L = 0.015, and p300 µg iAs/L = 0.023).

## Discussion

It is widely recognized that numerous sources of drinking water are contaminated with As, primarily due to natural emissions, human activities, and persistent recirculation [3]. The World Health Organization (WHO), along with the European Commission and the U.S. Environmental Protection Agency, has established a limit of 10 µg/L for As in drinking water. In several regions worldwide, including Bangladesh, Taiwan, Mexico, and parts of Southeast Asia, iAs concentrations in water sources exceed this guideline, posing serious public health risks [24–26].

Recent studies have linked chronic exposure to iAs concentrations as low as 10 µg/L with adverse health effects, including skin lesions, circulatory disorders, neurological and respiratory comorbidities, diabetes, and liver and renal dysfunctions [4, 27, 28]. Concentrations exceeding 50 µg iAs/L in drinking water have previously been associated with skin, lung, and bladder cancers, as well as sporadically with liver, kidney, and prostate cancers [29, 30]. Consequently, the doses utilized in this study include 10 µg iAs/L, 50 µg iAs/L, and a much higher concentration for which previous reports have suggested potential behavioral impairments [31].

Our results indicate that certain behavioral responses in zebrafish after chronic iAs exposure, assessed at Days 0, 7, 14, and 21, were subtle after 7 days, but significant changes in swimming patterns and social preference were observed at 50 µg iAs/L, particularly in the primary outcome of time spent in the left arm, suggesting a preference for the arm without social stimuli (Figure 5). Other endpoints, including total distance moved, velocity, and left arm entry frequency, were also statistically analyzed but should be interpreted as exploratory trends rather than definitive effects. Studies have suggested that the brain is a primary target for arsenic-induced toxicity [10, 32]. Both inorganic and organic As-based compounds have been shown to accumulate in various regions of the brain, indicating that As can easily traverse the BBB. Consequently, As accumulation in the brain may lead to structural changes in brain architecture and neurological disorders [32]. Furthermore, chronic exposure to heavy metals may increase the risk of neurodegeneration and cognitive impairments [33]. In this context, As intoxication has been associated with poor working memory and verbal comprehension, whereas lead is a well-documented neurotoxin, particularly concerning infantile neurotoxicity [10]. A recent study on As neurotoxicity in zebrafish larvae reported that exposure to 100 ppb As (equivalent to 100 µg/L) for 28 days resulted in reduced swimming distance, decreased swimming velocity, and diminished swimming time compared to non-exposed larvae. Another study noted significant reductions in locomotion parameters after 21 days of exposure to 100 - 300 ppb As (equivalent to 100–300 µg/L), suggesting a correlation between locomotion impairments and the neurotoxic effects of chronic As exposure [34].

Moreover, [35] reported significant manifestations of anxiety-like behavior only in the 100 ppb group. Our findings also suggest that adult zebrafish can exhibit anxiety-like behavioral patterns when exposed to lower concentrations of iAs for shorter durations (Figure 3C). Rachamalla et al. [36] reported an increased frequency of anxiety-like behaviors, as well as impaired locomotion and exploratory behavior, in an adult zebrafish model subjected to chronic dietary supplementation with arsenite over a 60-day period at concentrations of 30, 60, and 100 µg/g dry weight (d.w.). Additionally, this study proposes several mechanisms of arsenite toxicity, including correlations between motor dysfunction and decreased energy availability, oxidative stress, and dopaminergic dysfunction. The prolonged presence in the decision zone may reflect altered behavioral responses, while time spent in the starting arm could be associated with changes in behavioral performance.

Recent studies on As neurotoxicity indicate that learning and memory are significantly impaired due to disrupted neurogenesis and alterations in several key brain signaling pathways, including cholinergic, glutamatergic, and monoaminergic systems [37]. Notably, significant changes in hippocampal function, structure, and signaling have been correlated with cognitive deficits in a rat model of As neurotoxicity [38]. Despite methodological variations, most previous studies have reported neurotoxic effects consistent with the behavioral changes observed in our study. Rat model studies have elucidated mechanisms through which arsenic (As) exposure adversely affects learning, memory, and behavior. This impairment is linked to the disruption of glutamatergic signaling in the hippocampus and the Ras-mitogen-activated protein kinase (MAPK)/extracellular signal-regulated kinase (ERK) pathways [39]. In conjunction with reduced synaptic plasticity and altered neuronal structure, these models exhibited dose-dependent changes in motor activity, characterized by hyperactivity at low doses and hypoactivity at high doses [39]. Furthermore, in addition to cholinergic function disruption, As may also affect glucocorticoid signaling, resulting in increased stress responses and depressive-like behaviors [40]. In alignment with these findings, our data demonstrated a trend of hyperactivity at lower iAs doses during the 7–21 day monitoring period, while higher doses produced opposing effects (Figure 2A). Conversely, [31] reported that a 96-hour exposure to arsenic concentrations of 0.05 mg/L, 5 mg/L, and 15 mg/L resulted in contrasting locomotion patterns. Specifically, they observed significant decreases in locomotor activity in fish exposed to 5 mg As/L, while those exposed to 15 mg As/L exhibited significant hyperactivity, as evidenced by increased total swimming distance and velocity [31].

Similar studies on social preference have shown that exposures of 100–500 ppb As can impair locomotion and preference for the left arm of the apparatus (the arm with social stimuli) in a dose-dependent manner [35, 41]. Behavioral assessments revealed that zebrafish exposed to 500 ppb As exhibited increased angular velocity, a higher frequency of anxiety-like behaviors, and asynchrony in social interactions (e.g., out-of-sync circling movements with companions), although stereotypic repetitive behaviors were not observed. These findings suggest a potential relationship between As exposure, sociability, and anxiety. The authors propose mechanisms explaining the manifestation of social anxiety in As-exposed zebrafish, including oxidative stress and social motivation [35]. Additional studies have shown that embryonic exposure to arsenic trioxide does not immediately alter larval behavior but leads to delayed and persistent impairments in adulthood, such as reduced motor responses, loss of directional and color preference, and deficits in learning and cognitive flexibility [42]. Another study reported that exposed fish exhibited sensorimotor dysfunction and impaired habituation in startle response tests, along with disruptions in social interaction, locomotor activity, and associative learning during juvenile and adult stages [24].

In the present study, the observed increase in time spent in the decision zone and starting arm, coupled with altered locomotor and social preference patterns, aligns with prior findings. However, these must be interpreted cautiously due to the exploratory nature of some endpoints.

Under normal conditions, zebrafish can accumulate up to 10 µg As/g dry weight, a concentration significantly higher than that found in most terrestrial species (approximately 30 ng/g dry weight) [16]. Thus, zebrafish serve as excellent models for studying the neurotoxicological effects of As, while their heightened As uptake capacity presents future research opportunities, as it increases susceptibility to contamination and accumulation in the liver, kidneys, and brain [43]. Consequently, many critical physiological functions could be affected, including neurobehavior, nutrition, and reproduction, ultimately influencing ecological balance.

Regarding arsenic accumulation in zebrafish organs, this metalloid is absorbed from both water and diet and tends to concentrate in detoxification organs such as the liver and kidneys. Multiple studies have demonstrated that arsenic can accumulate in various adult tissues, explaining the organ-specific toxicity observed in fish. Similar to mammals, the liver serves as a primary site for arsenic metabolism, while other organs may also retain the metal, contributing to long-term toxic effects. This widespread distribution underscores the potential health risks for fish exposed to environmental arsenic and emphasizes the importance of monitoring arsenic levels in aquatic habitats [44]. Chronic dietary exposure to arsenic in zebrafish has been shown to impair reproduction and development in a sex-specific manner. Female fish exhibited reduced fecundity and alterations in egg quality, while males experienced changes in sperm function and decreased fertilization success. These reproductive deficits adversely affected embryonic development and offspring survival [45]. Other studies have indicated that arsenic is absorbed similarly, with the intestines serving as a primary site of uptake for arsenate (As(V)) rather than the gills. Limited biotransformation in zebrafish leads to significant bioaccumulation of inorganic arsenic in multiple organs, including the intestines, liver, kidneys, and muscle tissue. This widespread accumulation explains organ-specific toxicity and raises concerns for human health, particularly due to the retention of inorganic arsenic in edible tissues [46]. Natural organic matter, such as humic acid (HA), can influence arsenic bioaccumulation and biotransformation in aquatic organisms. In zebrafish, HA significantly reduced total arsenic accumulation during arsenite (As(III)) exposure, likely by forming a surface coating that hinders uptake, whereas this effect was not observed for arsenate (As(V)) exposure due to differences in uptake pathways. Arsenobetaine (AsB) was identified as the main biotransformation product, with HA enhancing its formation in arsenite exposure groups by decreasing levels of As(III) and monomethylarsonic acid (MMA), whereas no similar increase occurred during arsenate exposure. These findings underscore the role of environmental factors in modulating arsenic toxicity and accumulation, providing critical insights for assessing arsenic risks in freshwater ecosystems [47].

Studies investigating early developmental stages in zebrafish have demonstrated that arsenic exhibits pronounced toxicity and teratogenic effects. Exposure to sodium arsenite reduces survival at higher doses and induces morphological abnormalities, including spinal curvature, fin degeneration, pericardial and yolk sac edema, and reduced organ size. It also impairs behavioral responses and triggers oxidative stress, leading to altered antioxidant enzyme activities and upregulation of pro-inflammatory genes [48]. Furthermore, low concentrations of arsenic trioxide (As_2_O_3_) impair zebrafish development and behavior. Embryonic exposure reduces tail-coil movements, heartbeat, and swimming activity. Long-term effects include delayed hatching, reduced survival, and altered motor responses, along with deficits in directional and color preference in adults. Biochemical and metabolomic analyses indicate that arsenic trioxide disrupts proteins, lipids, and nucleic acids, while gene expression changes (upregulation of adsl and shank3a, downregulation of tsc1b) suggest interference with neural development pathways [42]. Even at environmentally relevant concentrations, exposure results in developmental malformations, including defects in body axis formation, pericardial edema, and reduced hatching success. These alterations are frequently accompanied by increased oxidative stress and DNA damage, further contributing to impaired growth and survival [49]. Collectively, these findings suggest that even low-level arsenic exposure can produce subtle yet significant effects on embryonic physiology, potentially heightening the risk of long-term metabolic or developmental disorders [50, 51].

Overall, these findings indicate that chronic As exposure leads to significant behavioral disruptions, particularly in primary social preference outcomes, while other locomotor and exploratory endpoints suggest exploratory trends. However, due to the single-tank design per treatment, pseudoreplication may limit the independence of fish-level data, necessitating cautious interpretation of exploratory endpoints. The absence of biochemical tissue measurements constitutes another limitation, preventing direct correlation between internal As levels and behavioral outcomes. Future studies that include multiple tanks per treatment and assess tissue accumulation would enhance the robustness of these conclusions.

## Conclusion

Our results demonstrate that a zebrafish model of chronic exposure to iAs exhibits distinct behavioral responses in a dose- and time-dependent manner for both locomotor performance and social preference tasks. These findings provide additional evidence that chronic iAs exposure can alter zebrafish behavior. Future research should focus on the significance of iAs contamination in both freshwater and marine ecosystems, its ecological impact, and potential implications for human health.
